# Supplementary material for: Causal Relationship between Aging and Anorexia Nervosa: A White-Matter-Microstructure-Mediated Mendelian Randomization Analysis
Source: Biomedicines. 2024 Aug 16;12(8):1874. doi: 10.3390/biomedicines12081874 (PMC11351342; doi:10.3390/biomedicines12081874)
Supplement: Supplementary file 1 [file biomedicines-12-01874-s001.zip › Supplementary Figure/Figure S4/Singe SNP plot of MR analysis for TL to ubm-b-1923.pdf]

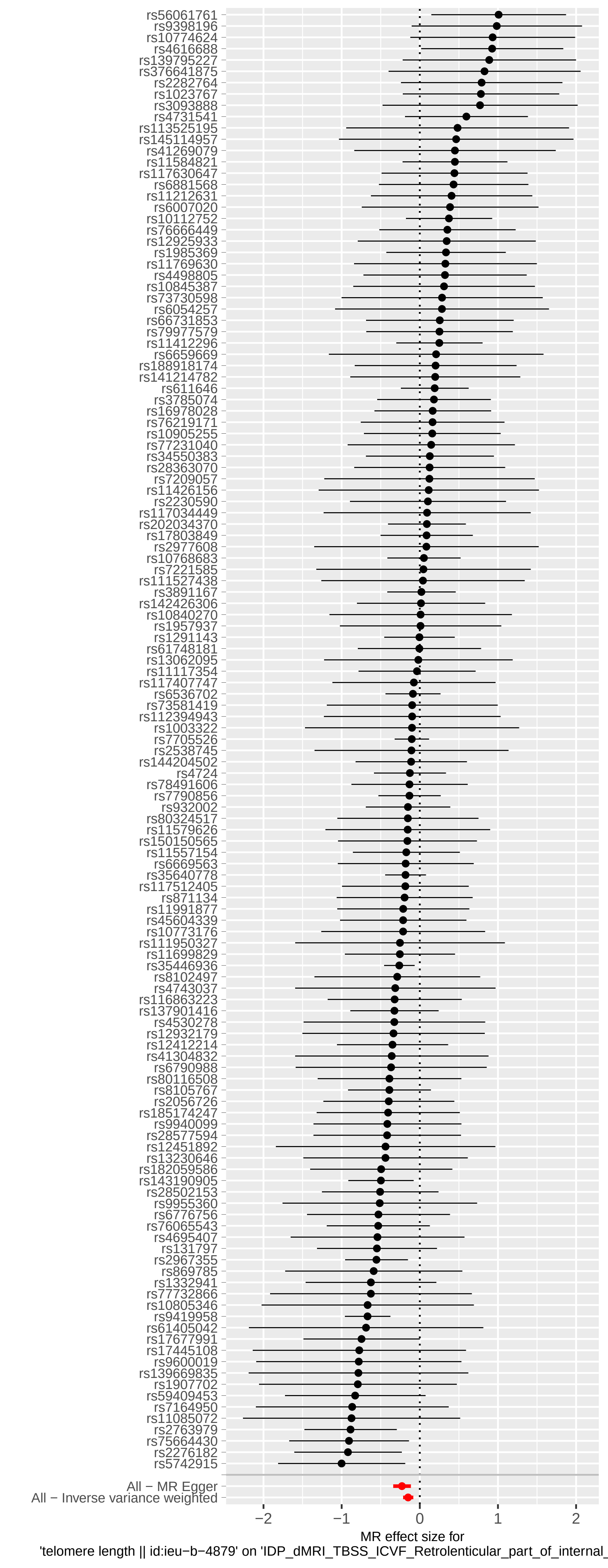
rs56061761
rs9398196
rs10774624
rs4616688
rs139795227
rs376641875
rs2282764
rs1023767
rs3093888
rs4731541
rs113525195
rs145114957
rs41269079
rs11584821
rs117630647
rs6881568
rs11212631
rs6007020
rs10112752
rs76666449
rs12925933
rs1985369
rs11769630
rs4498805
rs10845387
rs73730598
rs6054257
rs66731853
rs79977579
rs11412296
rs6659669
rs188918174
rs141214782
rs611646
rs3785074
rs16978028
rs76219171
rs10905255
rs77231040
rs34550383
rs28363070
rs7209057
rs11426156
rs2230590
rs117034449
rs202034370
rs17803849
rs2977608
rs10768683
rs7221585
rs111527438
rs3891167
rs142426306
rs10840270
rs1957937
rs1291143
rs61748181
rs13062095
rs11117354
rs117407747
rs6536702
rs73581419
rs112394943
rs1003322
rs7705526
rs2538745
rs144204502
rs4724
rs78491606
rs7790856
rs932002
rs80324517
rs11579626
rs150150565
rs11557154
rs6669563
rs35640778
rs117512405
rs871134
rs11991877
rs45604339
rs10773176
rs111950327
rs11699829
rs35446936
rs8102497
rs4743037
rs116863223
rs137901416
rs4530278
rs12932179
rs12412214
rs41304832
rs6790988
rs80116508
rs8105767
rs2056726
rs185174247
rs9940099
rs28577594
rs12451892
rs13230646
rs182059586
rs143190905
rs28502153
rs9955360
rs6776756
rs76065543
rs4695407
rs131797
rs2967355
rs869785
rs1332941
rs77732866
rs10805346
rs9419958
rs61405042
rs17677991
rs17445108
rs9600019
rs139669835
rs1907702
rs59409453
rs7164950
rs11085072
rs2763979
rs75664430
rs2276182
rs5742915
All − MR Egger
All − Inverse variance weighted
−2
−1
0
1
2
MR effect size for
'telomere length || id:ieu−b−4879' on 'IDP_dMRI_TBSS_ICVF_Retrolenticular_part_of_internal_
